# Long-Term Monitoring of Influenza A Viruses in Wild Waterfowl: Evidence from the Lake Baikal Basin (2018–2024)

**DOI:** 10.3390/v18070761

**Published:** 2026-07-11

**Authors:** Nikita Kasianov, Kirill Sharshov, Anastasiya Derko, Nikita Dubovitskiy, Junki Mine, Yuko Uchida, Evgeniya Badmaeva, Lopson Bazarov, Marina Gulyaeva, Arina Loginova, Maxim Grigoriev, Daria Kasianova, Tatiana Murashkina, Ivan Sobolev, Sachin Kumar, Wen Wang, Jianjun Chen, Alexander Shestopalov

**Affiliations:** 1 Federal Research Center of Fundamental and Translational Medicine, Novosibirsk 630060, Russia; sharshov@yandex.ru (K.S.); a.derko19@gmail.com (A.D.); nikitadubovitskiy@gmail.com (N.D.); mgulyaeva@gmail.com (M.G.); loginova995@gmail.com (A.L.); elerton70@gmail.com (M.G.); kasianovadari@yandex.ru (D.K.); murashkinatatiana89@gmail.com (T.M.); sobolev.riov@yandex.ru (I.S.); shestopalov2@mail.ru (A.S.); 2 Institute of Medicine and Medical Technologies, Novosibirsk State University, Novosibirsk 630090, Russia; 3 Division of Transboundary Animal Disease, National Institute of Animal Health, Tsukuba 305-0856, Japan; minejun84032@affrc.go.jp (J.M.); uchiyu@affrc.go.jp (Y.U.); 4 Institute of Natural Sciences, Dorji Banzarov Buryat State University, Ulan-Ude 670000, Russia; calidris03@gmail.com; 5 Federal State Budgetary Institution “Tunkinsky National Park”, Kyren 671010, Russia; lopson77@mail.ru; 6 Department of Biosciences and Bioengineering, Indian Institute of Technology Guwahati, Guwahati 781039, India; sachinku@iitg.ac.in; 7 State Key Laboratory of Plateau Ecology and Agriculture, Qinghai University, Xining 810016, China; 007cell@163.com; 8 State Key Laboratory of Virology and Biosafety, Wuhan Institute of Virology, Chinese Academy of Sciences, Wuhan 430071, China; chenjj@wh.iov.cn

**Keywords:** avian influenza virus, wild waterfowl, Lake Baikal, Buryatia, epidemiological surveillance, phylogenetic analysis, reassortment, Eurasian lineage, migratory flyways, full-genome sequencing

## Abstract

Wild waterfowl constitute the primary natural reservoir of influenza A viruses, and wetlands at the convergence of major migratory flyways serve as critical hubs for viral genetic exchange. Baikal Siberia, situated at the intersection of the East African–West Asian, Central Asian, and East Asian–Australasian flyways, represents a unique yet understudied region in this context. Here we report the results of long-term virological surveillance of wild birds in the Lake Baikal basin conducted between 2018 and 2024. A total of 1036 cloacal swab samples from 28 bird species were screened, yielding 42 influenza A virus isolates belonging to 12 HA/NA subtype combinations: H1N1, H3N1, H3N2, H3N5, H3N6, H3N8, H4N6, H6N1, H6N2, H6N3, H6N8, and H12N5. Among the detected subtypes, H6 viruses—identified with four distinct neuraminidase combinations (N1, N2, N3, N8)—are of particular public health relevance owing to their documented capacity for dual-receptor binding and potential for zoonotic transmission to mammals, including humans. Full-genome sequencing followed by cluster analysis of internal gene segments identified 16 distinct segment constellations, indicating extensive reassortment. BLAST searches against the GISAID database revealed closest genetic relatives in Mongolia, South Korea, Japan, China, and Western Siberia, with more distant links to Bangladesh, Europe, and a possible intercontinental connection via the Pacific flyway. Maximum-likelihood phylogenetic analysis of the HA and NA segments confirmed that all isolates belong to the Eurasian genetic lineage, yet they are distributed across multiple clades rather than forming a single monophyletic group, reflecting the role of Buryatia as a mixing zone for genetically diverse viral populations. These findings substantially expand the understanding of influenza A virus ecology in the Lake Baikal basin and underscore the importance of continued surveillance at this key migratory crossroads in Northern Asia.

## 1. Introduction

Influenza viruses are among the most extensively studied pathogens due to their substantial impact on both global public health and the economy. According to the International Committee on Taxonomy of Viruses (ICTV) [1], the family *Orthomyxoviridae* comprises the genera *Alphainfluenzavirus*, *Betainfluenzavirus*, *Deltainfluenzavirus*, and *Gammainfluenzavirus*. Among them, *Alphainfluenzavirus* is of particular epidemiological significance because of its ability to cause seasonal epidemics and global pandemics. Influenza A viruses infect a broad spectrum of hosts, including humans, horses, cattle, pigs, seals, and numerous avian species [2,3,4]. Among birds, it often causes epizootics, which result in severe economic losses to the poultry industry due to the mandatory culling of large numbers of farmed birds [5,6]. Wild waterfowl, as natural reservoirs of the influenza A virus, contribute to the spread of this pathogen over long distances, especially during seasonal migrations.

Baikal Siberia is a distinctive region of Eastern Siberia characterized by exceptional biodiversity and a strategic position at the intersection of major Eurasian bird migration routes. The central element of this ecosystem is Lake Baikal and its basin, including the Selenga River delta, which together form critically important habitats for migratory waterfowl and shorebirds.

Baikal Siberia features a unique combination of natural zones, ranging from mountain-taiga landscapes to steppe ecosystems. The Selenga River delta constitutes a particularly significant ecological area, rich in ecotones and diverse wetland habitats with varying topography, which supports high local avian species diversity [7]. According to ornithological studies, this area functions as a key staging and concentration zone for migratory birds, with numerous lakes and marshes serving as essential sites for the annual and biannual molt of wild ducks [8]. Extensive wetlands, particularly the Koymor wetlands, are located within the Tunka Depression situated on the southwestern flank of the Baikal Rift Zone [9].

A total of 149 species of waterbirds and shorebirds have been recorded in the Selenga delta, among which representatives of the orders Anseriformes and Charadriiformes predominate [7,10]. The delta wetlands are characterized by a dynamic hydrological regime that exerts a substantial influence on nesting bird density and habitat conditions [8].

The region serves as a convergence point for several major avian migratory flyways: the East African–West Asian Flyway, the Central Asian Flyway, and the East Asian–Australasian Flyway [7,11]. This is evidenced by the annual passage of millions of birds that use the Selenga River delta as a critical stopover, moulting, and breeding site [12,13]. Available data indicate high population dynamism: for example, the number of breeding ducks in the delta shows substantial interannual variation (up to 3–5-fold according to estimates from different years), and breeding density is directly dependent on water level and river discharge [12].

Hydrological changes in recent decades are characterized by biotic adaptation to the regulated water level of Lake Baikal; however, contemporary climate change is affecting intra-annual fluctuations in inflow and the phenology of bird residence [14]. The spatial design of field surveys is justified by the fact that the principal breeding and concentration areas of waterbird species shift continuously in response to the configuration of flooded habitats and lake level, necessitating regular monitoring across multiple sites within the delta [12]. The selection of sampling periods and key study sites is therefore directly informed by current data on hydrological cycles and the associated population dynamics.

The ecological conditions of the region create an optimal environment for virus circulation among wild birds. The aquatic ecosystems of the Baikal basin support waterfowl populations that constitute the primary natural reservoir of the influenza A virus gene pool [2]. The concentration of birds during migration and molt facilitates fecal–oral transmission of viruses through contaminated water, the principal mechanism of influenza virus spread among waterfowl [15].

Previous studies of influenza viruses in the Lake Baikal basin, although limited in number, have revealed considerable diversity among circulating strains. In September 2019, a monitoring study in the Lake Baikal basin, specifically in the Selenga River delta, analyzed 144 samples from wild birds [7]. Nine viruses were identified, yielding an overall viral prevalence of 6.25%: six belonged to the family *Orthomyxoviridae* (avian influenza viruses) and three to the family *Paramyxoviridae* (avian paramyxoviruses). Avian influenza viruses were isolated from mallard (*Anas platyrhynchos*) and gadwall (*Mareca strepera*). The following subtypes were identified: H6N8 from mallard, and H6N3, H3N8, and H12N5 from gadwall. Of particular interest is the H12N5 subtype, which is rarely isolated in nature and has a limited geographical distribution [16]. The H6 hemagglutinin strains identified in that study merit attention from a zoonotic perspective, as viruses of this subtype can exhibit affinity for mammalian receptors and potentially transmit to humans [17].

In September 2020, an expanded surveillance program collected 3018 fecal samples from wild migratory birds near Lake Baikal [18]. Samples were obtained from two principal sites: 1986 from the Selenga River delta and 1032 from the shore of Lake Arangatui, a satellite lake of Lake Baikal. Molecular screening identified 11 samples as positive for influenza A virus, and subsequent whole-genome sequencing determined the subtype as H13N8. The reference isolate was designated A/Unknown/Buryatia/Arangatui-1/2020 (H13N8). Although the exact host species could not be determined due to the fecal sampling approach, cormorants and gulls were the predominant bird species in the sampling area during the study period.

Taken together, prior surveillance efforts in the Lake Baikal basin have been limited in temporal coverage, restricted to one or two field seasons, and have not applied full-genome sequencing combined with systematic cluster and phylogenetic analysis across all gene segments. No study to date has described the multi-year dynamics of AIV subtype diversity and reassortment in this region using complete genomic data, leaving a substantial gap in our understanding of the role of Baikal Siberia in the intercontinental circulation of avian influenza viruses.

In the present study, we analyzed 1036 cloacal swab samples from 28 bird species collected between 2018 and 2024 in the Lake Baikal basin as part of ongoing epidemiological surveillance activities. The aim of this work was to characterize the genetic diversity of influenza A viruses circulating in wild waterfowl of the region through full-genome sequencing, cluster analysis of internal gene segments, and phylogenetic analysis of the external gene segments (*HA* and *NA*). Our findings contribute to the understanding of the role of this territory in the ecology and dissemination of avian influenza viruses along Eurasian migratory flyways.

## 2. Materials and Methods

### 2.1. Ethical Considerations

This study was conducted with the approval of the Biomedical Ethics Committee of the Federal Research Center of Fundamental and Translational Medicine of the Siberian Branch of the Russian Academy of Sciences (FRC FTM SB RAS), Novosibirsk (Protocols No. 2019-3 and 2021-10). Bird samples were collected during the hunting season under licenses issued by the regional Ministries of Ecology and Natural Resources as part of the annual biological material collection program (Infectious Diseases of Wild Animals Research Program, FRC FTM, Novosibirsk).

### 2.2. Sample Collection

Cloacal swabs from freshly harvested wild waterfowl were collected during the official hunting season into individual 2 mL tubes filled with 1 mL of glycerol-free viral transport medium consisting of phosphate-buffered saline (PBS, pH 7.5), amphotericin B (15 µg/mL), penicillin G (100 units/mL), and streptomycin (50 µg/mL). Tubes were immediately placed in liquid nitrogen and transported to the FRC FTM SB RAS laboratory for analysis [19]. Sample collection was carried out between 2018 and 2024 at sites with the highest bird densities in the Lake Baikal basin.

### 2.3. Virus Isolation Using Embryonated Chicken Eggs

For influenza A virus isolation, an aliquot was taken from each sample, vortexed, and centrifuged for 3 min at 3000× *g*. Antibiotics (penicillin and gentamicin) were added to the supernatant, which was then transferred to a new tube to prevent bacterial contamination. Subsequently, 100 µL of each sample was inoculated into the allantoic cavity of two specific pathogen-free (SPF) embryonated chicken eggs and incubated at 37 °C for 72 h in our Biosafety Level 3 (BSL-3) laboratory [20]. After incubation, 2 mL of allantoic fluid was collected from each egg and tested by hemagglutination assay (HA) using 5% chicken erythrocytes [21]. All HA-positive samples were aliquoted for PCR testing targeting the *M* gene of avian influenza virus. Three sequential passages in SPF embryonated chicken eggs were performed. Samples that did not exhibit hemagglutinating activity after three passages were considered negative.

### 2.4. RNA Extraction, Reverse Transcription, and PCR

All samples with HA activity were tested for the presence of influenza A virus. RNA was extracted from allantoic fluid using the RIBO-sorb kit (AmpliSens, Moscow, Russia). The extracted RNA was subjected to reverse transcription using the REVERTA-L kit (AmpliSens, Moscow, Russia). The presence of conserved regions of the influenza A virus M gene was determined by real-time PCR using the AmpliSens Influenza virus A/B-FL kit (AmpliSens, Moscow, Russia), designed to detect both human and avian influenza viruses.

### 2.5. Sequencing of Influenza A Viruses

The methodological approaches employed here are widely used for whole-genome influenza virus sequencing and downstream data analysis [22,23]. In our study, the initial virus isolation in SPF embryonated chicken eggs, subsequent PCR-based confirmation, and preparation of the isolate for sequencing indicate that the samples had a high viral titer (hemagglutination assay (*HA*) viral titer from 128 to 1024 HAU), allowing the preparation of high-quality libraries with a high proportion of viral reads by both multisegment amplification (M-RTPCR) library prep and RNA library construction.

For 2018–2021, we performed read filtering based on read mapping in CLC Genomics Workbench using the default Reads to Reference settings, including a minimum nucleotide count threshold of 2 and quality score-based consensus control. The output paired-end reads from the MiSeq second-generation sequencer (Illumina, San Diego, CA, USA) were mapped to reference sequences that were selected from a search of the Influenza Virus Database of the National Center for Biotechnology Information (NCBI) by using the FluGAS algorithm; this was followed by the construction of a consensus sequence when at least 3 reads were available. A single nucleotide was adopted when its representation exceeded 51% at that site, whereas mixed-base codes were adopted when multiple bases each accounted for at least 15% of the total coverage at the site. Processing of the short reads was performed following broadly accepted standard quality-control criteria: FastQC analysis, quality-trimming, short reads removing (<30), minimum coverage of 9× for consensus assembly (if less—ambiguous base (N)).

For complete genome sequencing of viruses collected in 2018, RNA was extracted from allantoic fluid using the GeneJET Viral DNA/RNA Purification Kit (Thermo Fisher Scientific, Waltham, MA, USA) and treated with TURBO DNase (Thermo Fisher Scientific). Up to 200 ng of RNA was used for cDNA library preparation with the NEBNext Ultra RNA Library Prep Kit (New England Biolabs, Ipswich, MA, USA). DNA libraries were sequenced using the Reagent Kit Version 3 (600-cycle) on the MiSeq genome sequencer (Illumina, San Diego, CA, USA) at the Genomics Core Facility (ICBFM SB RAS, Novosibirsk, Russia). Full-length genomes were assembled de novo using CLC Genomics Workbench v.9.5.3 (Qiagen, Hilden, Germany).

Viruses isolated between 2019 and 2021 were sequenced as part of a collaborative international program with the National Institute of Animal Health (Tsukuba, Japan). RNA was extracted from allantoic fluid using the RNeasy Mini Kit (Qiagen, Hilden, Germany). cDNA libraries were prepared using the NEBNext Ultra II RNA Library Prep Kit for Illumina (New England Biolabs, Ipswich, MA, USA). A total of 10 pM of libraries were mixed with 10 pM PhiX Control V3 (Illumina, San Diego, CA, USA) prior to sequencing. Sequencing was performed on the MiSeq genome sequencer (Illumina, San Diego, CA, USA) using the MiSeq Reagent Kit v.2 (Illumina, San Diego, CA, USA). Consensus sequences were assembled using CLC Genomics Workbench v.9.5.3 (Qiagen, Hilden, Germany).

Viruses isolated between 2022 and 2024 were sequenced on the Illumina MiSeq platform using the manufacturer’s reagent kits and protocols. RNA was extracted using the QIAamp Viral RNA Mini Kit (Qiagen, Hilden, Germany). Whole-genome amplification was performed following the modified protocol described by Zhou et al. [24]. DNA libraries were prepared using the Nextera DNA Flex Library Prep Kit (Illumina, San Diego, CA, USA). Sequencing was carried out on the MiSeq genome sequencer (Illumina, San Diego, CA, USA) using the Reagent Kit Version 3 (600-cycle). Raw Illumina reads were analyzed and assembled using the CFIA-NCFAD/nf-flu workflow (v3.2.1) [25], implemented on the Nextflow platform (v.24.04.2.5914) [26].

### 2.6. Search for the Nearest Identical Sequences for AIVs

The search for the most closely related sequences was performed using a local installation of BLAST+ (v2.16.0) [27] against the GISAID database [28].

### 2.7. Cluster Analysis of Internal Gene Segments

Sequences of internal gene segments from the viruses obtained in this study, as well as from viruses with complete genomes previously collected in the region and available in the GISAID and GenBank [29], international databases, were compiled into datasets for cluster analysis. Cluster analysis was performed in the R programming environment, version 4.5.1 [30] using hierarchical clustering with the UPGMA (Unweighted Pair Group Method with Arithmetic Mean) algorithm, corresponding to the “average” method in the hclust() function of the stats package [31]. Phylogenetic distance matrices were computed using the cophenetic correlation coefficient via the cophenetic.phylo() function from the ape package. For each internal segment, samples were assigned to four clusters using the cutree() function. The cluster color scheme was as follows: Cluster 1 (pink), Cluster 2 (blue), Cluster 3 (orange), and Cluster 4 (yellow).

### 2.8. Methodotology for Phylogenetic Analysis of External Gene Segments

Sequence datasets of the HA and NA segments from viruses obtained in this study, their closest BLAST matches were subjected to multiple sequence alignment using the MAFFT algorithm v7.520 (standalone version) [32] with default parameters (gap opening penalty: 1.53; gap extension penalty: 0.0). The resulting alignments were inspected in Unipro UGENE [33], where sites containing deletions were manually verified and removed from the final alignment.

Maximum-likelihood (ML) phylogenetic trees were constructed using the multithreaded version of IQ-TREE 2.3.6 for Linux x86 64-bit (built 4 August 2024) [34]. Branch support was assessed using the ultrafast bootstrap method, and the SH-aLRT test, each with 1000 replicates [35]. The optimal nucleotide substitution model for each gene segment was determined using ModelFinder according to the Bayesian Information Criterion (BIC) as implemented in IQ-TREE version 1.6 [36]. A complete list of selected models for all analyzed segments is provided in the Supplementary Materials (Table S2).

Tree visualization and topology analyses were performed in iTOL v.6 [37]. All trees were midpoint-rooted for display purposes.

### 2.9. Statistical Analysis

The 95% confidence intervals (CIs) for the proportion of AIV-positive samples were calculated using the exact Clopper-Pearson binomial method [38] implemented in R (version 4.5.1) [30].

## 3. Results

### 3.1. Ecological Features of Study Area and Samples

During this study, from 2018 to 2024, a total of 1036 cloacal swab samples from wild migratory birds belonging to six orders were collected and analyzed: Anseriformes, Charadriiformes, Gruiformes, Podicipediformes, Gaviiformes, and Suliformes (Table 1). The largest number of analyzed individuals belonged to the orders Anseriformes (731 of 1036; 70.56%), Gruiformes (200 of 1036; 19.31%), and Suliformes (76 of 1036; 7.34%). Information on the distribution of bird species by collection date, sample, and location details are provided in Table S1 in the Supplementary Materials.

**Figure 1:**
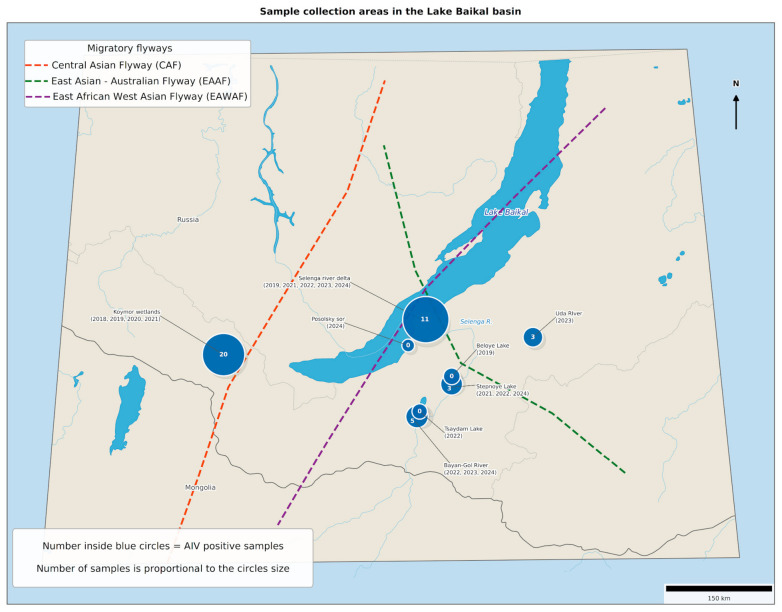
Sample collection areas in the Lake Baikal basin. For each site, the years of sampling and the number of samples testing positive for avian influenza virus (AIV) are indicated. The size of the blue circle is proportional to the total number of samples collected at that location.

Sampling was carried out across eight distinct sites within the Lake Baikal basin (Figure 1). At Koymor wetlands, samples were collected in 2018, 2019, 2020, and 2021, yielding a total of 392 samples, of which 20 tested positive for AIV. The Selenga river delta was sampled in 2019, 2021, 2022, 2023, and 2024, with 499 samples collected and 11 AIV-positive results. Beloye Lake was sampled in 2019 only, with 12 samples collected and no positive detections. Stepnoye Lake was sampled in 2021, 2022, and 2024, yielding 46 samples and 3 positive results. Bayan-Gol River was sampled in 2022, 2023, and 2024, with 50 samples collected and 5 AIV-positive results. Tsaydam Lake and Posolsky sor were each sampled during a single season (2022 and 2024, respectively), yielding 7 and 1 samples with no positive detections. Uda River was sampled in 2023, with 29 samples collected, of which 3 tested positive.

The highest numbers of AIV-positive samples were recorded at Koymor wetlands (n = 20) and the Selenga river delta (n = 11), which together accounted for 74% of all positive detections. Sites monitored across multiple seasons consistently yielded positive results, whereas single-season sites showed zero or low positivity, likely reflecting limited sampling effort rather than true absence of viral circulation. The spatial distribution of sampling sites and their annual AIV detection results are summarized in Figure 1.

### 3.2. Influenza A Viruses in Wild Birds of Buryatia

During the period from 2018 to 2024, virological monitoring in Buryatia yielded 42 strains of influenza A virus from 1036 collected samples, which were deposited in the GISAID international database. The isolates belonged to the following subtypes: H12N5, H1N1, H3N1, H3N2, H3N5, H3N6, H3N8, H4N6, H6N1, H6N2, H6N3, and H6N8 (Table 2). Forty-one viruses were isolated from the following representatives of the order Anseriformes: mallard (*Anas platyrhynchos*), common teal (*Anas crecca*), gadwall (*Mareca strepera*), northern pintail (*Anas acuta*), common pochard (*Aythya ferina*), Eurasian wigeon (*Mareca penelope*), and northern shoveler (*Spatula clypeata*). One virus was isolated from Eurasian coot *(Fulica atra*), a representative of the order Gruiformes.

### 3.3. Cluster Analysis and Nearest Sequences from the GISAID Database for Internal Genes

A summary table (Table 3) was constructed in which the assignment of each internal gene segment to a specific cluster is indicated by color. In addition, the sampling location of the nearest sequence identified by the BLAST algorithm from the GISAID international database is listed for each segment of each isolate.

Analysis of internal gene segments across all 42 isolates identified 16 distinct segment constellations (SC1–SC16), indicating extensive reassortment activity in the wild bird populations of the Lake Baikal basin. Three constellations predominated: SC3 (12 isolates, 2018–2023), SC10 (six isolates, 2018–2019), and SC1 (five isolates, 2019–2024), collectively accounting for 69% of all isolates. SC3 and SC10 are characterized by predominant genetic affinity with viruses from Mongolia and other Central Asian territories, suggesting a stable regional genomic backbone maintained through the Central Asian Flyway. SC1, by contrast, shows increasing representation of sequences from East Asia (China, South Korea, Japan) and South Asia (Bangladesh) in more recent samples, reflecting progressive introduction of eastern genetic material into the Baikal basin viral population. The remaining 13 constellations were each represented by one or two isolates and displayed more heterogeneous geographic origins, with links to Western Siberia, the Russian Far East, Europe (Sweden, The Netherlands, Georgia), the Middle East (Israel), and Southeast Asia (Vietnam), underscoring the role of the region as a crossroads for transcontinental viral gene flow. A detailed description of each constellation and its nearest BLAST matches is provided below.

Segment constellation 1 includes isolates A/Common Teal/Buryatia/73i/2019, A/mallard/Buryatia/Br55/2023, A/mallard/Buryatia/Br62/2023, A/common teal/Buryatia/Br18/2024, and A/mallard/Buryatia/Br52/2024, collected between 2019 and 2024. For all samples in this group, the nearest sequences for the majority of segments were found among isolates from Mongolia (2015–2019), predominantly from ducks, as well as from China (Shandong, Hunan, and Shanghai provinces; 2017–2022). Identity was also noted with Japanese isolates from ducks and swans (2013–2022), with viruses from Bangladesh obtained from environmental samples (2024), and with Russian viruses detected at Chany Lake, in Novosibirsk Region, Irkutsk Region, and Yakutia from mallards and teals (2005–2020). In individual cases, matches were identified with viruses from South Korea, Vietnam, and Kazakhstan. The temporal range of reference sequences spans 2005–2024.

Segment constellation 2 is represented by isolate A/mallard/Buryatia/Br70/2023. Its segment sequences show high identity with viruses from South Korea (2019) isolated from mallards, from China (Hunan Province, 2019) from wild birds, from Japan (2016) from ducks, from Bangladesh (2024) from environmental samples, and from Sweden (2024) from great black-backed gull.

Segment constellation 3 comprises isolates A/pochard/Buryatia/33/2018, A/Common Teal/Buryatia/89i/2019, A/Gadwall/Buryatia/2221/2019, A/mallard/Buryatia/25i/2020, A/european wigeon/Buryatia/26i/2020, A/common teal/Buryatia/56i/2020, A/mallard/Buryatia/127z/2021, A/gadwall/Buryatia/160z/2021, A/common teal/Buryatia/164z/2021, A/mallard/Buryatia/176z/2021, A/shoveler/Buryatia/B78/2022, and A/mallard/Buryatia/Br52/2023, isolated between 2018 and 2023. For the majority of these, the most closely related sequences were found among viruses from Mongolia (2015–2022), China (Shandong, Jiangxi, Anhui, and Liaoning provinces; 2016–2019), South Korea (2019–2021), and Russian regions (Novosibirsk Region, Chany Lake, Primorsky Krai, and Amur Region). Identity was also noted with isolates from Bangladesh, Georgia, The Netherlands, Japan, and Sweden. The temporal range of reference sequences spans 2013–2024.

Segment constellation 4 consists of isolates A/Mallard/Buryatia/44i/2019 and A/Gadwall/Buryatia/2226/2019 (both from 2019). For A/Mallard/Buryatia/44i/2019, the majority of segments are most closely related to viruses from ducks in Mongolia (2015–2019), from Chany Lake (2018), and from mallards in South Korea (2019). For the second isolate, matches were found with viruses from Primorsky Krai (2019) from mallards, from Mongolia (2019) from ducks, from Amur Region (2020) from teals, from South Korea (2019) from mallards, and from Bangladesh (2019) from ducks.

Segment constellation 5 includes isolates A/northern pintail/Buryatia/94i/2020, A/gadwall/Buryatia/B10/2021, A/mallard/Buryatia/B57/2021, and A/mallard/Buryatia/Br81/2023, collected between 2020 and 2023. Their segment sequences show predominant relatedness to viruses from South Korea (2019–2021), China (Jiangxi, Shandong, and Shanghai provinces; 2016–2023), and Russian regions (Amur Region, Chany Lake, and Dagestan). Additional sources include Bangladesh (2019–2024), Sweden (2021), and Japan (2022). For A/mallard/Buryatia/Br81/2023, the MP segment showed a link to a virus isolated from a human in China (2023).

Segment constellation 6 is formed by A/teal/Buryatia/63/2018 and A/Gadwall/Buryatia/2209/2019. The first isolate is closely related to viruses from Egypt (2017) from northern shoveler, from Russia (Chany Lake, 2016–2018) from mallards and gadwall, from The Netherlands (2018) from mallards and teals, and from Kazakhstan (2018) from northern shoveler. A/Gadwall/Buryatia/2209/2019 demonstrates high identity with viruses from Mongolia (2018–2019).

Segment constellation 7 contains A/mallard/Buryatia/B47/2021 and A/mallard/Buryatia/B122/2022, whose segment sequences are characterized by links to Mongolian isolates (2015–2018) and viruses from Bangladesh (2024). For isolate B122/2022, identity of the NP segment with a virus from South Korea isolated in 2019 was also noted.

Segment constellation 8 is represented by isolates A/common teal/Buryatia/150z/2021 and A/common teal/Buryatia/B149/2022. The nearest sequences for their segments were found in South Korea, Taiwan, Mongolia, Vietnam, China (Hunan Province), Russia (Novosibirsk Region, 2018; Amur Region, 2020), Japan, and Bangladesh. The PB2 and NS segments in this case belong to Clusters 2 and 3, respectively, and show identity to the same viruses.

Segment constellation 9 consists solely of A/gadwall/Buryatia/B12/2021, whose segment sequences demonstrate mixed origin: segments are closely related to isolates from South Korea (2022), Russia (Sakhalin and Amur Region; 2020), Japan (2021), and Egypt (2005).

Segment constellation 10 includes isolates A/teal/Buryatia/57/2018, A/teal/Buryatia/100/2018, A/common teal/Buryatia/22i/2019, A/Common Teal/Buryatia/88i/2019, A/Gadwall/Buryatia/2206/2019, and A/Gadwall/Buryatia/2252/2019, isolated in 2018–2019. For the majority of these, matches were found with Mongolian viruses (2015–2019), with some samples nearly completely matching isolate A/duck/Mongolia/401/2018. Individual segments are closely related to viruses from Bangladesh (2024), Japan (2016), and South Korea (2020). The segment sequences of A/Gadwall/Buryatia/2206/2019 and A/Gadwall/Buryatia/2252/2019 showed identity with viruses collected in Omsk Region (2019), Sakhalin (2020), South Korea (2019), Georgia (2018), Amur Region (2020), China (Shanghai, 2019), Bangladesh (2019), and Mongolia (2018).

The internal gene segment sequences of A/northern pintail/Buryatia/B11/2021, forming Segment constellation 11, demonstrate links to isolates from China (Shandong Province, 2017; Jiangxi, 2016), South Korea (2019), Japan (2007, 2015), and Sweden (2024).

Segment constellation 12 comprises A/mallard/Buryatia/114/2018, A/Eurasian coot/Buryatia/12i/2020, and A/common teal/Buryatia/B151/2022. The closest identity matches for their segments originate from Mongolia (2015–2019), China (Jiangxi and Shandong provinces; 2017–2020), South Korea (2021), Taiwan (2018), Vietnam (2020), Bangladesh (2024), and Russia (Novosibirsk Region, 2018; Amur Region, 2020).

Segment constellation 13, formed by virus A/mallard/Buryatia/1575/2019, has segments whose sequences are most closely related to viruses from Mongolia (2007, 2015), Japan (2013, 2016), Russia (Amur Region, 2020), and China (Shandong Province, 2019).

Segment constellation 14 is represented by isolate A/duck/Buryatia/664-5465/1988, obtained from the GISAID international database and originally isolated in 1988. Its segment sequences show the greatest identity with viruses also isolated in the previous century, between 1961 and 1987, in South Africa, Japan, Germany, Hong Kong, and Taiwan. This segment constellation also exhibits the greatest diversity of cluster assignments.

Segment constellation 15 (A/Unknown/Buryatia/Arangatui-1/2020) is characterized by close relatedness to H13 subtype viruses isolated from gulls. The nearest sequences by percent identity were found in South Korea (2021), The Netherlands (2013), and Belgium (2020).

Segment constellation 16 includes isolate A/pochard/Buryatiya/1941/2000. Its segment sequences are most closely related to those of viruses from Mongolia (2001), Japan (1999, 2000, 2008), Taiwan (1998), and a 1961 mallard isolate.

### 3.4. Phylogenetic Analysis of External Gene Segments

Maximum-likelihood phylogenetic trees were constructed for the external gene segments of the studied viruses. As the surface glycoproteins HA and NA are the main determinants of pathogenicity and are antigenically relevant for vaccines, we analyzed the HA and NA segments of subtypes H12N5, H1N1, H3N1, H3N2, H3N5, H3N6, H3N8, H4N6, H6N1, H6N2, H6N3, and H6N8 detected in the Baikal basin region. Phylogenetic analysis of the HA and NA segments of all 12 subtype combinations detected in this study revealed that the Buryatian isolates consistently belong to the Eurasian genetic lineage of avian influenza viruses. Notably, they do not form a single monophyletic group but are instead distributed across multiple independent clades, reflecting the role of the Lake Baikal basin as a mixing zone for genetically diverse viral populations. A characteristic feature of the investigation strains is their frequent basal position on the phylogenetic trees—observed for H1, H12, N1, N2, N3, and N5 segments—which may indicate early or ancestral circulation of these variants in the region prior to their dispersal along Eurasian flyways. Genetic links were identified with viruses from a broad geographic range, spanning Western Siberia and Europe in the west to Mongolia, China, Japan, South Korea, and Bangladesh in the east, with an isolated possible intercontinental connection suggested by the clustering of 2023 N8 isolates with a virus from Alaska. Detailed cladistic descriptions have been retained in the main text for subtypes H3N8 and H4N6 (which account for the largest number of isolates, n = 14 and n = 11 respectively) and for H12N5; the latter, despite the small number of isolates (n = 2), is of particular interest due to its limited global distribution and the basal phylogenetic position of the studied strains compared to other Eurasian and South Asian representatives of this subtype. Phylogenetic trees for the remaining subtypes are provided in the Supplementary Materials (Figures S1–S5).

**Figure 2:**
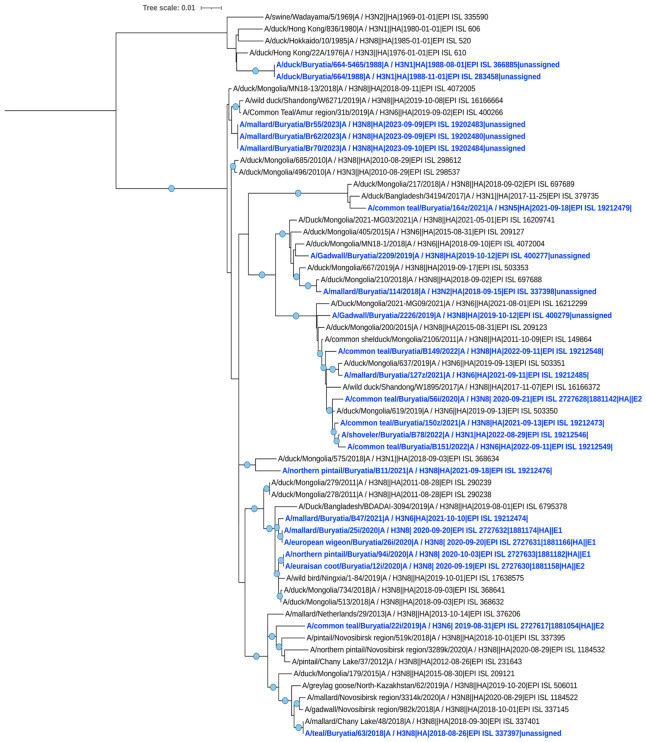
Maximum likelihood phylogenetic tree of the HA (H3) genome segment of avian influenza viruses isolated in the Lake Baikal basin. The tree is midpoint-rooted. The blue circle symbol denotes branches with values SH-aLRT > 80% and UFboot > 95%. The studied strains are highlighted in blue. The tree scale represents the number of substitutions per site.

For the HA segment of subtype H3 (Figure 2), the studied isolates belong to the Eurasian genetic lineage of avian influenza viruses. Two archival isolates from 1988 (A/duck/Buryatia/664/1988 and A/duck/Buryatia/664-5465/1988), obtained from the GISAID international database and originally collected in the Buryatia Region (of Russia), demonstrate relatedness to viruses from China and Japan also isolated in the late twentieth century. The 2023 isolates (A/mallard/Buryatia/Br55, Br62, and Br70) form a compact cluster with viruses from Amur Region and Shandong Province (China), indicating a link to the East Asian migratory system.

A substantial proportion of Buryatian isolates from 2020 to 2022 cluster together with Mongolian viruses, forming a Central Asian cluster. Individual Buryatian samples (A/teal/Buryatia/63/2018, A/common teal/Buryatia/22i/2019) are associated with viruses from Western Siberia (Novosibirsk Region, Chany Lake), while isolate A/common teal/Buryatia/164z/2021 (H3N5) groups with viruses from Bangladesh and Mongolia, occupying a distinct position on the tree. Overall, the diversity of phylogenetic groupings among Buryatian H3 strains reflects the role of the region as a mixing zone for viral populations of diverse geographical origins.

**Figure 3:**
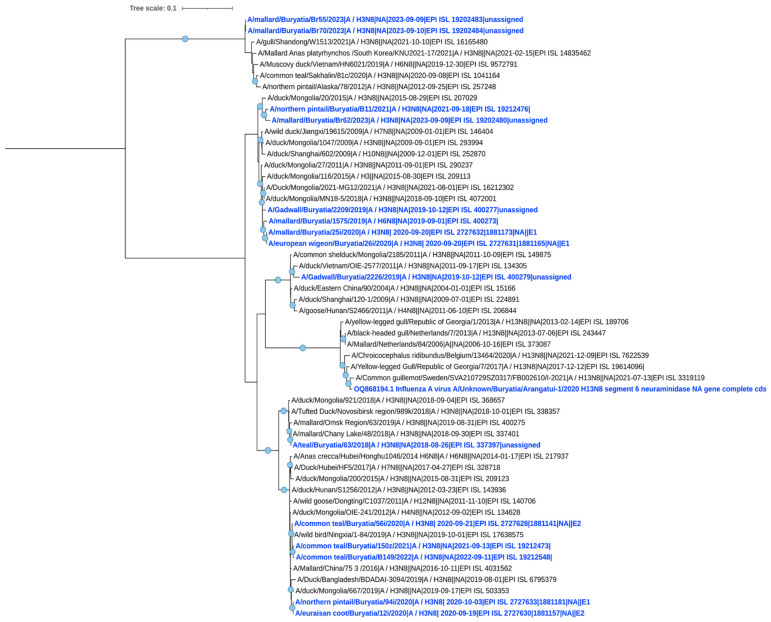
Maximum likelihood phylogenetic tree of the NA (N8) genome segment of avian influenza viruses isolated in the Lake Baikal basin. The tree is midpoint-rooted. The blue circle symbol denotes branches with values SH-aLRT > 80% and UFboot > 95%. The studied strains are highlighted in blue. The tree scale represents the number of substitutions per site.

For the NA segment of subtype N8 (Figure 3), the studied viruses also belong to the Eurasian lineage of avian influenza viruses. Isolates A/Gadwall/Buryatia/2209/2019 and A/mallard/Buryatia/1575/2019 (H6N8) cluster with viruses from Mongolia, while A/european wigeon/Buryatia/26i/2020 and A/mallard/Buryatia/25i/2020 are nearly identical to each other. Isolates from 2020 to 2022 (B149, 150z, 56i, 12i, 94i) fall within a large clade together with Mongolian and Chinese viruses. Isolate A/teal/Buryatia/63/2018 groups with a virus from Chany Lake, forming a Western Siberian cluster.

The 2023 isolates (A/mallard/Buryatia/Br55, Br62, and Br70) form a compact cluster that is sister to a group of viruses with broad geographical coverage, including isolates from Alaska, Sakhalin, Vietnam, and South Korea, suggesting a possible connection to the Pacific migratory flyway.

A/Unknown/Buryatia/Arangatui-1/2020 (H13N8) forms a separate clade with isolates collected in European countries, isolated from gulls.

**Figure 4:**
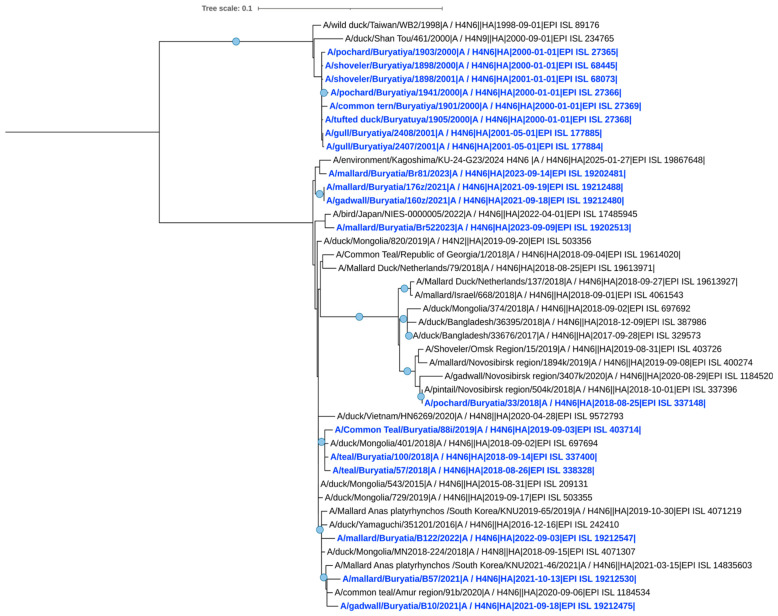
Maximum likelihood phylogenetic tree of the HA (H4) genome segment of avian influenza viruses isolated in the Lake Baikal basin. The tree is midpoint-rooted. The blue circle symbol denotes branches with values SH-aLRT > 80% and UFboot > 95%. The studied strains are highlighted in blue. The tree scale represents the number of substitutions per site.

For the HA segment of subtype H4 (Figure 4), the Buryatian isolates belong to the Eurasian genetic lineage and are represented in two temporal groups. Early strains from 2000 to 2001 (A/shoveler/Buryatiya/1898, A/pochard/Buryatiya/1903, A/pochard/Buryatiya/1941, and others), obtained from the GISAID international database and isolated from northern shovelers, common pochards, tufted ducks, gulls, and common terns, form a distinct cluster with minimal intragroup divergence, indicating intensive local circulation of a single virus variant among various species of waterfowl and shorebirds.

Modern Buryatian isolates (2018–2023) are distributed across several clades. Isolates A/gadwall/Buryatia/160z/2021 and A/mallard/Buryatia/176z/2021 are closely related to an isolate from Japan (Kagoshima, 2024), and A/mallard/Buryatia/Br81/2023 falls within the same subcluster. Isolate A/pochard/Buryatia/33/2018 groups with viruses from Novosibirsk and Omsk Regions, while isolates from 2021 to 2022 (B57, B10, B122) are associated with viruses from Mongolia, South Korea, and Amur Region. This distribution underscores the sustained connection of Buryatian H4 virus populations with both Western Siberian and East Asian genetic lineages over more than two decades.

**Figure 5:**
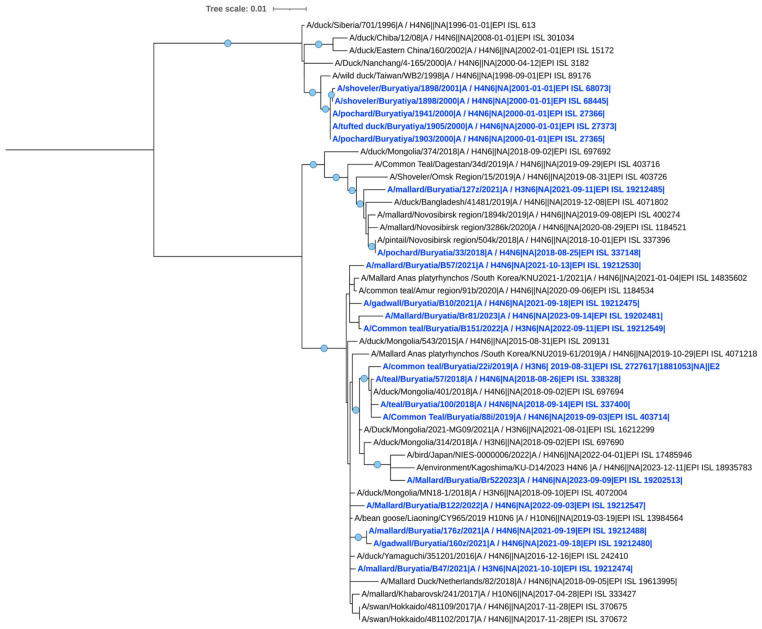
Maximum likelihood phylogenetic tree of the NA (N6) genome segment of avian influenza viruses isolated in the Lake Baikal basin. The tree is midpoint-rooted. The blue circle symbol denotes branches with values SH-aLRT > 80% and UFboot > 95%. The studied strains are highlighted in blue. The tree scale represents the number of substitutions per site.

The NA segment sequences of subtype N6 (Figure 5) belong to the Eurasian genetic lineage of avian influenza viruses. Early strains from 2000 to 2001 (H4N6), obtained from the GISAID international database and isolated from northern shovelers, common pochards, and tufted ducks, form a distinct historical cluster most closely related to viruses from Taiwan (1998) and Siberia (1996). Modern Buryatian isolates (2018–2023) are distributed across several groupings: A/pochard/Buryatia/33/2018 and A/mallard/Buryatia/127z/2021 are associated with viruses from Western Siberia and Dagestan; isolates from 2021 to 2023 (B151, Br81, B10, B57) cluster with Mongolian and South Korean strains; and isolates A/gadwall/Buryatia/160z/2021 and A/mallard/Buryatia/176z/2021 are linked to Chinese viruses. The N6 segment was found in combination with HA subtypes H3 and H4, confirming ongoing neuraminidase gene reassortment in wild bird populations of Buryatia.

**Figure 6:**
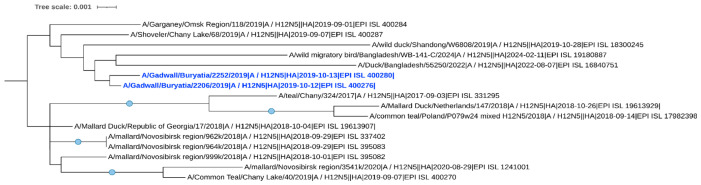
Maximum likelihood phylogenetic tree of the HA (H12) genome segment of avian influenza viruses isolated in the Lake Baikal basin. The tree is midpoint-rooted. The blue circle symbol denotes branches with values SH-aLRT > 80% and UFboot > 95%. The studied strains are highlighted in blue. The tree scale represents the number of substitutions per site.

For the HA segment of subtype H12 (Figure 6), the Buryatian isolates belong to the Eurasian genetic lineage of avian influenza viruses. Two samples—A/Gadwall/Buryatia/2206/2019 and A/Gadwall/Buryatia/2252/2019, isolated from gadwall one day apart (12–13 October 2019)—occupy a basal position under midpoint rooting, forming a compact group with a high degree of nucleotide similarity. The remaining viruses are divided into two subgroups. The first includes isolates from Western Siberia (Novosibirsk Region, Omsk Region, Chany Lake) and the Georgia, dated 2018–2020. The second subgroup unites viruses from Bangladesh (2022 and 2024), which demonstrate the greatest genetic distance from the other members of the dataset. The basal position of the Buryatian strains relative to the Western Siberian and South Asian isolates may indicate early circulation of this H12 variant among wild waterfowl in Central Asia.

**Figure 7:**
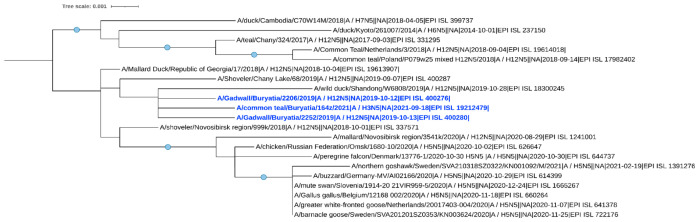
Maximum likelihood phylogenetic tree of the NA (N5) genome segment of avian influenza viruses isolated in the Lake Baikal basin. The tree is midpoint-rooted. The blue circle symbol denotes branches with values SH-aLRT > 80% and UFboot > 95%. The studied strains are highlighted in blue. The tree scale represents the number of substitutions per site.

For the NA gene of subtype N5 (Figure 7), three Buryatian isolates belong to the Eurasian genetic lineage. Isolates A/Gadwall/Buryatia/2206/2019 and A/Gadwall/Buryatia/2252/2019 (both H12N5) form a group together with isolate A/common teal/Buryatia/164z/2021 (H3N5), occupying a basal position on the tree. The main clade includes viruses from Western Siberia (Novosibirsk Region, Chany Lake), Georgia, as well as H5N5 isolates associated with highly pathogenic avian influenza epizootics in Europe (Sweden, The Netherlands, Belgium, Slovenia, Germany, Denmark) and Russia (Omsk Region) in 2020–2021. The presence of a shared N5 segment in viruses of different HA subtypes (H3, H5, H6, H7, H12) provides evidence of extensive reassortment and underscores the contribution of wild birds of Buryatia to the dissemination of influenza virus genetic material along Eurasian flyways.

## 4. Discussion

The Lake Baikal basin constitutes a complex system of wetlands that plays a critical role in supporting waterfowl populations of Eastern Siberia. The Selenga River delta, the largest wetland in the basin, encompasses a network of channels, oxbow lakes, floodplain meadows, and marshes forming a mosaic of habitats of varying productivity [39]. The delta serves as an important site for nesting, molting, and staging of numerous waterfowl species. The coastal zones of Lake Baikal include a system of lowland marshes formed by tributaries and flooded floodplains; however, the lake itself provides relatively few suitable habitats for waterfowl due to characteristics of its shoreline, depth, and limited shallow-water areas [39].

During our surveillance conducted between 2018 and 2024, complete genomes of 42 influenza A viruses were obtained from 1036 samples collected from wild migratory birds representing 28 species belonging to 6 orders.

The annual distribution of samples was uneven: the highest numbers were collected in 2019 (n = 245) and 2023 (n = 189), whereas 108 samples were collected in 2018, 104 in 2020, 139 in 2021, 171 in 2022, and only 80 in 2024.

Most samples were obtained from representatives of the order Anseriformes (731 out of 1036; 70.56%). Moreover, 41 out of the 42 isolated viruses came from this order: 13 cases in Eurasian teal (A), 15 in mallard, and single positive samples in gadwall, Eurasian wigeon, northern shoveler, northern pintail, and common pochard. This is consistent with the fact that the main reservoirs of avian influenza A viruses are birds of this order [2].

Among Anseriformes, the dominant species by sample size were mallard (*Anas platyrhynchos*, n = 160), Eurasian teal (*Anas crecca*, n = 124), and gadwall (*Mareca strepera*, n = 115)—all widespread species.

Substantial material was also obtained from the Eurasian coot—a representative of the order Gruiformes (200 out of 1036; 19.31%)—but only one sample of this species tested positive.

The orders Charadriiformes (n = 16), Podicipediformes (n = 9) and Gaviiformes (n = 1) were represented by much smaller sample sizes, which does not allow reliable conclusions about the role of these birds in epizootiology.

Samples from the order Suliformes (79 out of 1036; 7.62%), represented by a single species—the great cormorant—deserve special mention. The great cormorant has rapidly increased its abundance at the border of its range after almost half a century of absence from Lake Baikal due to prolonged droughts in Central Asia, and has redistributed within its historical range, forming thousands of colonies on islands, bays, shallows (sors), and along the shores of Lake Baikal and adjacent lake systems [40,41]. We collected 79 samples from this species. None of them tested positive for AIV (0%, 95% CI: 0–4.56%). However, the potential risks of AIV transmission and spread, combined with the large population size, indicate the need for continued monitoring.

Overall, the observed infection rate (4.05%) corresponds to moderately low epizootic activity in the region during 2018–2024. However, the wide confidence intervals for many species highlight the need to increase sample sizes to refine risk assessment.

The subtype diversity encompassed 12 distinct HA/NA combinations. Some of these, such as H3N8 and H4N6, are widely distributed in wild bird populations [42,43,44], whereas others, such as H12N5 and H6N3, are rarely encountered.

Avian influenza viruses of the H6Nx subtype pose a significant threat to public health. They are widely distributed among wild and domestic birds and are capable of crossing species barriers, infecting pigs, dogs, and humans [45]. Research findings indicate that a substantial proportion of circulating H6 strains (up to 34% in some live poultry market samples) have already acquired the ability to bind human-type receptors (α-2,6-linked sialic acids) while retaining affinity for avian-type receptors [17]. Key amino acid substitutions in hemagglutinin, such as E190V, G228S, Q226L, and P186L, play a critical role in this shift in receptor specificity, enabling these viruses to replicate efficiently in mammalian lungs without prior adaptation [46].

At the same time, H12 subtype viruses, although detected much less frequently, also exhibit characteristics that increase their epidemiological significance [47]. Although most H12 strains retain a preference for avian-type receptors, recent studies have identified isolates with dual binding specificity for both avian and human receptors [48]. The ability of Eurasian reassortants, such as the SX/2143 (H12N2) strain, to efficiently infect the lungs of mice without prior adaptation indicates the presence of latent risks to human health [49]. Although some H12N5 variants currently exhibit low replication efficiency in mammalian models [50], the high frequency of intercontinental reassortment among H12 viruses warrants enhanced global epidemiological surveillance of this “rare” subtype.

Cluster analysis and BLAST searches of internal gene segments revealed a complex pattern of genetic diversity and allowed us to establish probable evolutionary links with isolates from extensive geographical zones of Eurasia. The studied strains exhibited a high degree of genetic heterogeneity across their internal segments and were assigned to 16 conditional segment constellations, indicating active reassortment in natural bird populations. Analysis of the nearest BLAST matches for each of the six internal segments (PB2, PB1, PA, NP, MP, and NS) yielded the following principal observations.

The sequences of segments of the studied viruses are predominantly of Eurasian origin. The closest relationships were traced to isolates circulating in East Asia. A substantial number of segments showed the highest identity with sequences detected in Mongolia, underscoring the importance of this region as a source of genetic diversity for viruses circulating in southern Siberia. Numerous links with isolates from China, South Korea, and Japan were also identified.

An important finding is the detection of distant genetic connections. A number of segments, particularly the MP gene, demonstrated high similarity with viruses isolated in Bangladesh, in some cases with very recent samples (2024). Matches were also recorded with isolates from Europe (Sweden, The Netherlands, Georgia), the Middle East (Israel), and Southeast Asia (Vietnam). These findings point to transcontinental transfer of genetic material, likely mediated by migratory birds along flyway corridors.

A degree of segment-specific variation was observed. For example, the PB2 and PA segments were frequently most closely related to Mongolian isolates of the H4N6 subtype, whereas the MP gene characteristically showed links to viruses from Bangladesh (H7N7). At the same time, different samples within a single segment constellation may exhibit distinct relatedness patterns for individual genes, which may reflect their reassortant nature.

Links were demonstrated between avian influenza viruses isolated in Buryatia and those from key breeding territories of wild migratory birds such as the Republic of Yakutia. Research conducted there [42] revealed widespread prevalence and circulation of various influenza virus subtypes in wild migratory bird populations and possible transcontinental transmission. In the context of the Central Asian Flyway, which traverses both territories, this finding particularly underscores the importance of continued research and monitoring.

Phylogenetic analysis of external genes (HA and NA) of influenza A viruses isolated from wild birds in the Lake Baikal basin also demonstrated that all studied isolates belong to the Eurasian genetic lineage. Notably, the Buryatian strains do not form a single monophyletic group but are instead distributed across multiple clades, demonstrating genetic links with viruses from diverse geographical regions of Eurasia—from Western Siberia and Europe to Mongolia, East Asia, and South Asia. A characteristic feature of the Buryatian isolates is their frequent basal position on the phylogenetic trees (segments H1, H12, N1, N2, N3, N5), which may indicate early circulation of ancestral virus variants in this territory. Concurrently, a number of samples cluster with genetically distant strains: the 2024 H1 isolates are associated with both Western Eurasian (Georgia, Sweden, Belgium) and East Asian (Primorsky Krai, South Korea) viruses; the 2023 N8 samples are linked to a clade that includes a virus from Alaska, suggesting possible intercontinental transfer of genetic material.

It should be noted that the previously published genomic characterization of A/Unknown/Buryatia/Arangatui-1/2020 (H13N8) [18] provides a valuable regional perspective, while the present study extends this work through a broader methodological framework. Specifically, our analysis incorporated GISAID—the primary global repository for influenza genome sharing—enabling a more comprehensive representation of global viral diversity. Furthermore, whereas the phylogenetic reconstruction in that study focused on sequences of the same subtype, our approach encompassed the full spectrum of available genetic lineages for each genomic segment, irrespective of HA/NA combination. This strategy is particularly important for the robust identification of reassortment events, as restricting comparisons to a single subtype may not capture the full scope of inter-lineage genetic exchange. Inclusion of a broader set of waterfowl lineages from GISAID in the present study allowed for a more comprehensive reconstruction of the evolutionary relationships among circulating viruses. Notably, the detection of extensive intra-subtype reassortment among H13N8 influenza viruses reported by those authors remains a noteworthy finding, and our results further corroborate the role of the Baikal Siberia region as a potential hotspot for genetic exchange between viral lineages [51].

In conclusion, the need for further research in the Lake Baikal basin should be emphasized. Given the limited number of studies conducted in this region to date, such work is of critical importance for monitoring and forecasting the epidemiological situation, as well as for understanding the significance of Buryatia from both ornithological and epidemiological perspectives.

## 5. Limitations of the Study

### 5.1. Sampling Strategy

Study limitations concerning the bias from single cloacal swab sampling and lack of serological surveillance should be noted. We recognize the limitation of our study related to single cloacal swab sampling. The tasks of the multi-year monitoring conducted by our institute include the assessment of the genetic diversity of viruses relevant for a particular season, the assessment of molecular markers of pathogenicity, the isolation of isolates and their transfer to store at the state collection/reference centers. Most of the tasks are related to the study of current viruses circulating in specific populations, which allows timely assessment of phylogenetic relationships and events related to the exchange of segments. Collecting blood from birds involves additional serious technical difficulties, logistics and costs for reagents and transportation (often several thousand kilometers), as well as the need for special skills to collect blood. In addition, ELISA reagent kits developed for poultry may not be suitable for all bird species [52]. The development of a special ELISA reagent kit for Anseriformes is also associated with different tasks and was not included in the study.

### 5.2. Isolation and Sequencing Strategy

The isolation of RNA viruses in bird populations is uneven. Internal and external factors make populations heterogeneous for virus replication, which leads to different viral loads. Studies have shown that 20% of individuals in the population may be responsible for 80% of viral transmission [53,54]. When collecting samples from wild birds, there is a possibility that these superspreaders will not be included in our sample. To minimize this probability, we try to increase the number of individuals in the sample. However, even at the design stage of the study, based on information about the average percentage of AIV release in wild bird populations (according to available sources) and the uneven viral load in the population, it was decided to increase the number of studied individuals. At the same time, by reducing the number of samples that we collect from 1 individual. We recognize that this is a limitation of our research and should be noted.

Another issue is a strategy chosen that first involves isolation in embryonated eggs and then further analysis of isolates using PCR. Some studies showed the loss of sensitivity suggesting that the RT-qPCR is the most sensitive method. Additionally, even if no isolate can be obtained from a PCR-positive sample, sequence data can usually still be generated. Indeed, when conducting epidemiological surveillance, the Real-time RT-PCR method in the primary sample is the most traditional and preferable (WOAH) to identify the genetic material of the virus. However, isolating isolates in embryonated eggs is the most appropriate option for detecting a virus capable of infecting cells and replicating. The purpose of the long-term monitoring of influenza A virus conducted by our institute is not only to identify the genetic material and subtyping, but also to determine the biological properties and transfer new isolates to the state collection/reference center. Especially if HPAIV is detected.

There are a number of studies comparing the virus isolation method on chicken embryos and PCR. A number of works indicate a greater sensitivity of PCR diagnostics (for example, Kim et al., 2019) [55], others indicate a higher sensitivity of isolation in chicken embryos (for example, Spackman et al., 2003) [56]. The results on average show that they can be compared in sensitivity. Obviously, separate methods of detection have some limitations. Therefore, different methods should be combined for optimal surveillance. We chose VI method with further PCR and sequencing because we tried not only to detect but also to isolate the virus so that we could then have sufficient concentration and sequence the complete genome. It is much more difficult to sequence the complete genome from the original specimens because they could usually contain highly degraded RNA. Previous studies indicate that, when the viral load in primary samples is low, the likelihood of recovering a full-genome viral sequence is reduced [57,58]. At the same time, we recognize an important limitation of detection after isolation: virus isolation rates are lower than molecular RT-PCR rates; mixed infections may be lost during cultivation, which may affect the assessment of influenza virus subtype diversity; genetic mutations may be introduced via AIV propagation in chicken embryos. Finally, after considering all limitations and benefits of different strategies, it was decided to cultivate the virus before sequencing.

The use of several sequencing workflows in a long-term surveillance study has some limitations. The use of different library preparation protocols and assembly workflows was due to the longitudinal nature of the study and to the fact that sequencing was performed in different years and, for part of the material, within an international collaborative program.

Because the surveillance program covered several years and part of the sequencing was performed within an external collaborative framework, the original raw sequencing files are not available to the author group for all historical samples. Therefore, we cannot retrospectively apply a fully uniform raw-read-level QC pipeline to all sequencing batches or provide standardized read-depth and read-filtration statistics for every sample. However, since different library preparation protocols, reagent kits, and assembly workflows were used in different years, all newly generated sequences in this study were obtained using the same sequencing platform, Illumina MiSeq. The differences therefore mainly concerned the upstream library preparation/amplification procedures and downstream assembly workflows, rather than the sequencing technology itself. Given the small size of the segmented influenza A virus genome and the use of consensus genome sequences for downstream analysis, we consider the resulting sequences to be comparable for the purposes of subtype identification, BLAST-based similarity search, segment-level phylogenetic reconstruction, and operational clustering of internal gene segments.

The analyses performed in this study were intentionally restricted to the consensus-genome level. We did not perform analyses that require strict read-level comparability across sequencing batches, such as minor-variant detection, intra-host diversity analysis, low-frequency SNP profiling, or quantitative assessment of within-sample variant frequencies. Thus, the possible technical differences between sequencing batches are unlikely to affect the main conclusions of the manuscript, which are based on robust whole-segment relationships rather than read-level variation. The consensus-level comparative analysis of influenza A virus sequences generated by different sequencing platforms and library preparation protocols is a standard practice in influenza molecular epidemiology. Public influenza databases such as GISAID contain sequences generated in different laboratories using different sequencing technologies, including Sanger sequencing and various next-generation sequencing platforms, as well as different amplification and library preparation protocols. These data are routinely used together for subtype assignment, BLAST comparison, and phylogenetic analysis, provided that the submitted consensus sequences are complete or near-complete, biologically plausible, and pass sequence-level quality checks. Our study follows the same consensus-level logic. Although this issue should be taken into account when providing comparative studies.

## 6. Conclusions

The data obtained demonstrate that the influenza A virus population in Baikal Siberia constitutes a dynamic and genetically heterogeneous system in constant interaction with viruses from extensive territories across Asia and Europe. Virus circulation is maintained through the regular introduction of new genetic variants by migratory birds and subsequent local reassortment. The strategic location of the region at the intersection of major Eurasian avian migratory flyways, combined with the presence of key ornithological territories such as the Selenga River delta, creates optimal conditions for the circulation, evolution, and emergence of novel reassortant influenza A virus variants.

These findings substantially expand our understanding of the molecular epidemiology of influenza A virus in Baikal Siberia and contribute to knowledge of viral evolution and dissemination in Northern Asia. The results underscore the necessity of continuous epidemiological surveillance at this important flyway node for understanding the global evolution of influenza A virus. Continued systematic monitoring will enable timely detection of emerging highly pathogenic strains and assessment of the risks they pose to wild and domestic bird populations and to public health.

## Figures and Tables

**Table 1:** Sample size and results of virus detection in wild waterfowl of the Lake Baikal basin.

Order	Species	2018	2019	2020	2021	2022	2023	2024	Total Number	Number of AIV	Positivity Rate (CI 95%)
Anseriformes (*n* = 731)	eurasian teal (*Anas crecca*)	27	21	27	17	15	6	11	124	13	10.48% (5.7–17.26%)
	mallard (*Anas platyrhynchos*)	15	22	10	31	28	33	21	160	15	9.38% (5.34–14.99%)
	gadwall (*Mareca strepera*)	14	46	11	17	3	24	0	115	8	6.96% (3.05–13.25%)
	tufted duck (*Aythya fuligula*)	10	31	25	9	2	18	0	95	0	0% (0–3.81%)
	common goldeneye (*Bucephala clangula*)	5	14	3	18	0	2	2	44	0	0% (0–8.04%)
	eurasian wigeon (*Mareca penelope*)	2	8	9	0	1	4	8	32	1	3.13% (0.08–16.22%)
	northern shoveler (*Spatula clypeata*)	10	9	0	0	4	1	3	27	1	3.7% (0.09–18.97%)
	northern pintail (*Anas acuta*)	2	0	10	2	4	6	2	26	2	7.69% (0.95–25.13%)
	common pochard (*Aythya ferina*)	4	7	3	2	7	8	0	31	1	3.23% (0.08–16.7%)
	garganey (*Spatula querquedula*)	12	5	1	3	1	3	0	25	0	0% (0–13.72%)
	smew (*Mergellus albellus*)	0	1	0	2	1	10	6	20	0	0% (0–16.84%)
	ruddy shelduck (*Tadorna ferruginea*)	0	2	0	2	11	0	0	15	0	0% (0–21.8%)
	bean goose (*Anser fabalis*)	0	0	0	1	0	0	1	2	0	0% (0–84.19%)
	common merganser (*Mergus merganser*)	0	0	0	0	0	7	0	7	0	0% (0–40.96%)
	red-breasted merganser (*Mergus serrator*)	0	2	0	1	1	0	0	4	0	0% (0–60.24%)
	falcated duck (*Mareca falcata*)	2	0	0	0	0	0	0	2	0	0% (0–84.19%)
	mandarin duck (*Aix galericulata*)	0	0	0	0	1	0	0	1	0	0% (0–97.5%)
	baikal teal (*Sibirionetta formosa*)	0	0	0	0	1	0	0	1	0	0% (0–97.5%)
Charadriiformes (*n* = 16)	spotted redshank (*Tringa erythropus*)	0	0	0	0	7	0	0	7	0	0% (0–40.96%)
	ruff (*Calidris pugnax*)	0	6	0	0	0	0	0	6	0	0% (0–45.93%)
	common redshank (*Tringa totanus*)	0	0	0	0	0	0	2	2	0	0% (0–84.19%)
	common gull (*Larus canus*)	0	1	0	0	0	0	0	1	0	0% (0–97.5%)
Gruiformes (*n* = 200)	eurasian coot (*Fulica atra*)	1	66	4	31	72	19	6	199	1	0.5% (0.01–2.77%)
	baillon’s crake (*Zapornia pusilla*)	1	0	0	0	0	0	0	1	0	0% (0–97.5%)
Podicipediformes (*n* = 9)	great crested grebe (*Podiceps cristatus*)	1	2	0	1	1	2	0	7	0	0% (0–40.96%)
	black-necked grebe (*Podiceps nigricollis*)	0	0	1	1	0	0	0	2	0	0% (0–84.19%)
Gaviiformes (*n* = 1)	black-throated loon (*Gavia arctica*)	1	0	0	0	0	0	0	1	0	0% (0–97.5%)
Suliformes (*n* = 79)	great cormorant (*Phalacrocorax carbo*)	1	2	0	1	11	46	18	79	0	0% (0–4.56%)
6 orders	28 species	108	245	104	139	171	189	80	1036	42	4.05%

**Table 2:** The strains of AIV isolated during the study.

Virus Name	Subtype	Host	Collection Date	ID GISAID
A/pochard/Buryatia/33/2018	H4N6	common pochard (*Aythya ferina)*	25 August 2018	337148
A/teal/Buryatia/57/2018	H4N6	common teal (*Anas crecca*)	26 August 2018	338328
A/teal/Buryatia/63/2018	H3N8	common teal (*Anas crecca*)	26 August 2018	337397
A/teal/Buryatia/100/2018	H4N6	common teal (*Anas crecca*)	14 September 2018	337400
A/mallard/Buryatia/114/2018	H3N2	mallard (*Anas platyrhynchos*)	15 September 2018	337398
A/common teal/Buryatia/22i/2019	H3N6	common teal (*Anas crecca*)	31 August 2019	2727617
A/Mallard/Buryatia/44i/2019	H6N1	mallard (*Anas platyrhynchos*)	1 September 2019	403705
A/mallard/Buryatia/1575/2019	H6N8	mallard (*Anas platyrhynchos*)	1 September 2019	400273
A/Common Teal/Buryatia/73i/2019	H6N1	common teal (*Anas crecca*)	1 September 2019	403713
A/Common Teal/Buryatia/89i/2019	H1N1	common teal (*Anas crecca*)	3 September 2019	403715
A/Common Teal/Buryatia/88i/2019	H4N6	common teal (*Anas crecca*)	3 September 2019	403714
A/Gadwall/Buryatia/2226/2019	H3N8	gadwall (*Mareca strepera*)	12 October 2019	400279
A/Gadwall/Buryatia/2221/2019	H6N3	gadwall (*Mareca strepera*)	12 October 2019	400278
A/Gadwall/Buryatia/2209/2019	H3N8	gadwall (*Mareca strepera*)	12 October 2019	400277
A/Gadwall/Buryatia/2206/2019	H12N5	gadwall (*Mareca strepera*)	12 October 2019	400276
A/Gadwall/Buryatia/2252/2019	H12N5	gadwall (*Mareca strepera*)	13 October 2019	400280
A/eurasian coot/Buryatia/12i/2020	H3N8	eurasian coot (*Fulica atra*)	19 September 2020	2727630
A/eurasian wigeon/Buryatia/26i/2020	H3N8	eurasian wigeon (*Mareca penelope*)	20 September 2020	2727631
A/mallard/Buryatia/25i/2020	H3N8	mallard (*Anas platyrhynchos*)	20 September 2020	2727632
A/common teal/Buryatia/56i/2020	H3N8	common teal (*Anas crecca*)	21 September 2020	2727628
A/northern pintail/Buryatia/94i/2020	H3N8	northern pintail (*Anas acuta*)	3 October 2020	2727633
A/mallard/Buryatia/127z/2021	H3N6	mallard (*Anas platyrhynchos*)	11 September 2021	19212485
A/common teal/Buryatia/150z/2021	H3N8	common teal (*Anas crecca*)	13 September 2021	19212473
A/gadwall/Buryatia/B10/2021	H4N6	gadwall (*Mareca strepera*)	18 September 2021	19212475
A/gadwall/Buryatia/B12/2021	H6N2	gadwall (*Mareca strepera*)	18 September 2021	19212472
A/northern pintail/Buryatia/B11/2021	H3N8	northern pintail (*Anas acuta*)	18 September 2021	19212476
A/common teal/Buryatia/164z/2021	H3N5	common teal (*Anas crecca*)	18 September 2021	19212479
A/gadwall/Buryatia/160z/2021	H4N6	gadwall (*Mareca strepera*)	18 September 2021	19212480
A/mallard/Buryatia/176z/2021	H4N6	mallard (*Anas platyrhynchos*)	19 September 2021	19212488
A/mallard/Buryatia/B47/2021	H3N6	mallard (*Anas platyrhynchos*)	10 October 2021	19212474
A/mallard/Buryatia/B57/2021	H4N6	mallard (*Anas platyrhynchos*)	13 October 2021	19212530
A/shoveler/Buryatia/B78/2022	H3N1	northern shoveler (*Spatula clypeata*)	29 August 2022	19212546
A/mallard/Buryatia/B122/2022	H4N6	mallard (*Anas platyrhynchos*)	3 September 2022	19212547
A/common teal/Buryatia/B149/2022	H3N8	common teal (*Anas crecca*)	11 September 2022	19212548
A/common teal/Buryatia/B151/2022	H3N6	common teal (*Anas crecca*)	11 September 2022	19212549
A/mallard/Buryatia/Br62/2023	H3N8	mallard (*Anas platyrhynchos*)	9 September 2023	19202480
A/mallard/Buryatia/Br55/2023	H3N8	mallard (*Anas platyrhynchos*)	9 September 2023	19202483
A/mallard/Buryatia/Br52/2023	H4N6	mallard (*Anas platyrhynchos*)	9 September 2023	19202513
A/mallard/Buryatia/Br70/2023	H3N8	mallard (*Anas platyrhynchos*)	10 September 2023	19202484
A/mallard/Buryatia/Br81/2023	H4N6	mallard (*Anas platyrhynchos*)	14 September 2023	19202481
A/common teal/Buryatia/Br18/2024	H1N1	common teal (*Anas crecca*)	20 September 2024	20146001
A/mallard/Buryatia/Br52/2024	H1N1	mallard (*Anas platyrhynchos*)	3 October 2024	20146000

**Table 3:** Cluster assignment of each internal gene segment indicated by color. Cluster 1 (pink), Cluster 2 (blue), Cluster 3 (orange), Cluster 4 (yellow). Samples sharing the same cluster-based segment composition are grouped into conditional segment constellations (SC), as indicated in the corresponding column.

Virus	PB2	PB1	PA	NP	MP	NS	Segment Constellation	Subtype
73i/2019	Mongolia	Mongolia	Mongolia	Chany Lake	Mongolia	Shanghai	SC1	H6N1
Br55/2023	Shandong	Hunan	Mongolia	Yamaguchi	Bangladesh	Mongolia		H3N8
Br62/2023	Shandong	Kagoshima	Mongolia	Yamaguchi	Bangladesh	Irkutsk		H3N8
Br18/2024	Shandong	Novosibirsk region	South Korea	Bangladesh	Bangladesh	Novosibirsk region		H1N1
Br52/2024	Saga	South Korea	North-Kazakhstan	Vietnam	Bangladesh	Yakutia		H1N1
Br70/2023	South Korea	Hunan	Yakutia	Yamaguchi	Bangladesh	Sweden	SC2	H3N8
33/2018	Chany Lake	Novosibirsk region	Novosibirsk region	Bangladesh	Georgia	The Netherlands	SC3	H4N6
89i/2019	Primorsky Krai	Mongolia	Mongolia	Mongolia	Mongolia	Mongolia		H1N1
2221/2019	Mongolia	Hunan	Mongolia	Mongolia	South Korea	Bangladesh		H6N3
25i/2020	South Korea	Amur region	Mongolia	Anhui	Chany Lake	Jiangxi		H3N8
26i/2020	South Korea	Amur region	Mongolia	Anhui	Chany Lake	Jiangxi		H3N8
56i/2020	South Korea	Chany Lake	Mongolia	Anhui	Chany Lake	South Korea		H3N8
127z/2021	Primorsky Krai	South Korea	Georgia	Mongolia	Bangladesh	Novosibirsk region		H3N6
160z/2021	Novosibirsk region	Shandong	Chany Lake	Chany Lake	Bangladesh	Novosibirsk region		H4N6
164z/2021	South Korea	South Korea	Liaoning	Bangladesh	Chany Lake	Novosibirsk region		H3N5
176z/2021	South Korea	Shandong	Chany Lake	Chany Lake	Bangladesh	Novosibirsk region		H4N6
B78/2022	Novosibirsk region	Israel	Novosibirsk region	Novosibirsk region	Novosibirsk region	Shanghai		H3N1
Br52/2023	Shandong	South Korea	Sweden	Aichi	Bangladesh	Jiangxi		H4N6
44i/2019	Mongolia	Mongolia	Mongolia	Chany Lake	Mongolia	South Korea	SC4	H6N1
2226/2019	Primorsky Krai	Mongolia	Amur region	South Korea	Mongolia	Bangladesh		H3N8
94i/2020	South Korea	Amur region	Jiangxi	Mongolia	Chany Lake	South Korea	SC5	H3N8
B10/2021	South Korea	Amur region	Amur region	Amur region	South Korea	Amur region		H4N6
B57/2021	South Korea	South Korea	Shandong	Jiangxi	Bangladesh	Shanghai		H4N6
Br81/2023	Chany Lake	Dagestan	Shandong	Sweden	Zhejiang	Jiangxi		H4N6
63/2018	Egypt	Chany Lake	Chany Lake	The Netherlands	North-Kazakhstan	The Netherlands	SC6	H3N8
2209/2019	Mongolia	Mongolia	Mongolia	Mongolia	Mongolia	Mongolia		H3N8
B47/2021	Mongolia	Mongolia	Mongolia	Mongolia	Bangladesh	Mongolia	SC7	H3N6
B122/2022	South Korea	Mongolia	Mongolia	South Korea	Bangladesh	Mongolia		H4N6
150z/2021	South Korea	Yunlin	Mongolia	Vietnam	Hunan	Novosibirsk region	SC8	H3N8
B149/2022	Amur region	Yunlin	Okayama	Mongolia	Bangladesh	Novosibirsk region		H3N8
B12/2021	South Korea	Sakhalin	Amur region	Gifu	South Korea	Egypt	SC9	H6N2
57/2018	Mongolia	Mongolia	Mongolia	Mongolia	Bangladesh	Yamaguchi	SC10	H4N6
100/2018	Mongolia	Mongolia	Mongolia	Mongolia	Mongolia	Mongolia		H4N6
22i/2019	Mongolia	Mongolia	Mongolia	Mongolia	South Korea	Mongolia		H3N6
88i/2019	Mongolia	Mongolia	Mongolia	Mongolia	Mongolia	Mongolia		H4N6
2206/2019	Omsk Region	Mongolia	Amur region	South Korea	Sakhalin	South Korea		H12N5
2252/2019	Georgia	Mongolia	Amur region	Bangladesh	Mongolia	Shanghai		H12N5
B11/2021	Shandong	South Korea	Chiba	Jiangxi	Hokkaido	Sweden	SC11	H3N8
114/2018	Mongolia	Mongolia	Mongolia	Mongolia	Mongolia	Mongolia	SC12	H3N2
12i/2020	Mongolia	Mongolia	Jiangxi	Mongolia	Shandong	South Korea		H3N8
B151/2022	Amur region	Yunlin	Amur region	Vietnam	Bangladesh	Novosibirsk region		H3N6
1575/2019	Mongolia	Saga	Amur region	Shandong	Fukuoka	Mongolia	SC13	H6N8
664-5465/1988	South Africa	Shimane	Potsdam	Hong Kong	Taiwan	South Africa	SC14	H3N1
Arangatui-1/2020	South Korea	South Korea	South Korea	The Netherlands	Belgium	South Korea	SC15	H13N8
1941/2000	Mongolia	Hokkaido	Shimane	Taiwan	Russian Federation	Chiba	SC16	H4N6

## Data Availability

All genome sequences of AIVs from this study are available in the GISAID database. (accession numbers: EPI ISL 337148, EPI ISL 338328, EPI ISL 337397, EPI ISL 337400, EPI ISL 337398, EPI ISL 2727617, EPI ISL 403705, EPI ISL 400273, EPI ISL 403713, EPI ISL 403715, EPI ISL 403714, EPI ISL 400279, EPI ISL 400278, EPI ISL 400277, EPI ISL 400276, EPI ISL 400280, EPI ISL 2727630, EPI ISL 2727631, EPI ISL 2727632, EPI ISL 2727628, EPI ISL 2727633, EPI ISL 19212485, EPI ISL 19212473, EPI ISL 19212475, EPI ISL 19212472, EPI ISL 19212476, EPI ISL 19212479, EPI ISL 19212480, EPI ISL 19212488, EPI ISL 19212474, EPI ISL 19212530, EPI ISL 19212546, EPI ISL 19212547, EPI ISL 19212548, EPI ISL 19212549, EPI ISL 19202480, EPI ISL 19202483, EPI ISL 19202513, EPI ISL 19202484, EPI ISL 19202481, EPI ISL 20146001 and EPI ISL 20146000).

## Supplementary Materials

The following supporting information can be downloaded at https://www.mdpi.com/article/10.3390/v18070761/s1: Table S1: Sample size and results of virus detection with year-wise distribution in wild waterfowl of the Lake Baikal basin. Table S2: The optimal nucleotide substitution model for each gene segment, selected using the ModelFinder program based on the Bayesian Information Criterion (BIC). Figure S1: Maximum likelihood phylogenetic tree of the HA (H6) genome segment of avian influenza viruses isolated in the Lake Baikal basin. The tree is midpoint-rooted. The blue circle symbol denotes branches with values SH-aLRT > 80% and UFboot > 95%. The studied strains are highlighted in blue. The tree scale represents the number of substitutions per site. Figure S2: Maximum likelihood phylogenetic tree of the NA (N1) genome segment of avian influenza viruses isolated in the Lake Baikal basin. The tree is midpoint-rooted. The blue circle symbol denotes branches with values SH-aLRT > 80% and UFboot > 95%. The studied strains are highlighted in blue. The tree scale represents the number of substitutions per site. Figure S3: Maximum likelihood phylogenetic tree of the NA (N2) genome segment of avian influenza viruses isolated in the Lake Baikal basin. The tree is midpoint-rooted. The blue circle symbol denotes branches with values SH-aLRT > 80% and UFboot > 95%. The studied strains are highlighted in blue. The tree scale represents the number of substitutions per site. Figure S4: Maximum likelihood phylogenetic tree of the NA (N3) genome segment of avian influenza viruses isolated in the Lake Baikal basin. The tree is midpoint-rooted. The blue circle symbol denotes branches with values SH-aLRT > 80% and UFboot > 95%. The studied strains are highlighted in blue. The tree scale represents the number of substitutions per site. Figure S5: Maximum likelihood phylogenetic tree of the HA (H1) genome segment of avian influenza viruses isolated in the Lake Baikal basin. The tree is midpoint-rooted. The blue circle symbol denotes branches with values SH-aLRT > 80% and UFboot > 95%. The studied strains are highlighted in blue. The tree scale represents the number of substitutions per site.
