# Supplementary figures and images for: Long-Term Monitoring of Influenza A Viruses in Wild Waterfowl: Evidence from the Lake Baikal Basin (2018–2024)
Source: Viruses. 2026 Jul 11;18(7):761. doi: 10.3390/v18070761 (PMC13431582; doi:10.3390/v18070761)

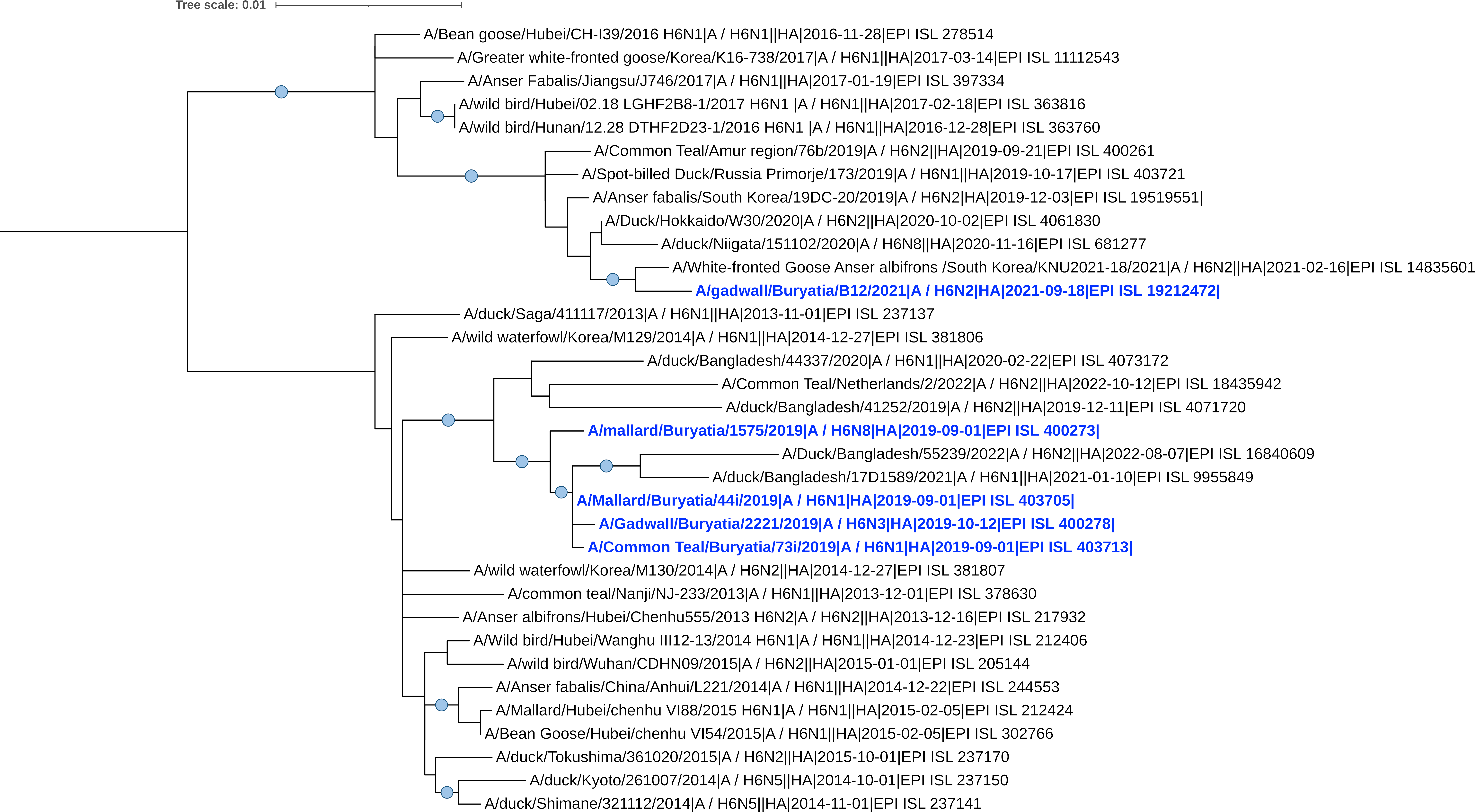

Supplement: Supplementary file 1 [file viruses-18-00761-s001.zip › Figure S1.png]

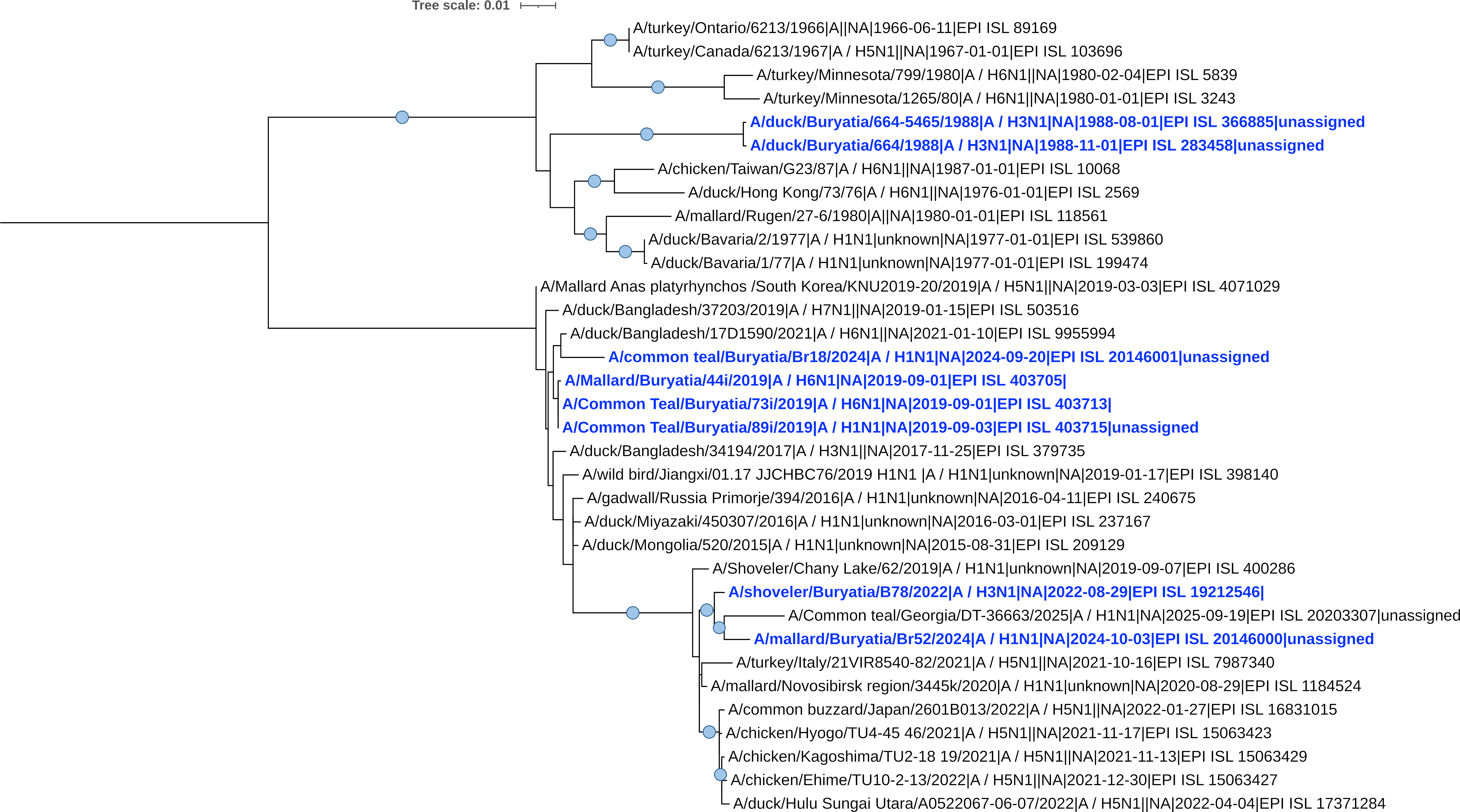

Supplement: Supplementary file 1 [file viruses-18-00761-s001.zip › Figure S2.png]

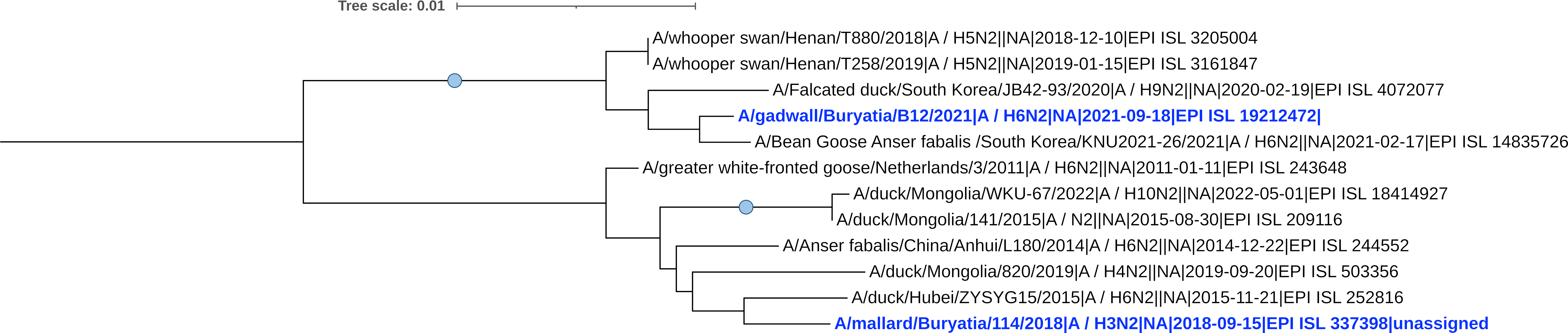

Supplement: Supplementary file 1 [file viruses-18-00761-s001.zip › Figure S3.png]

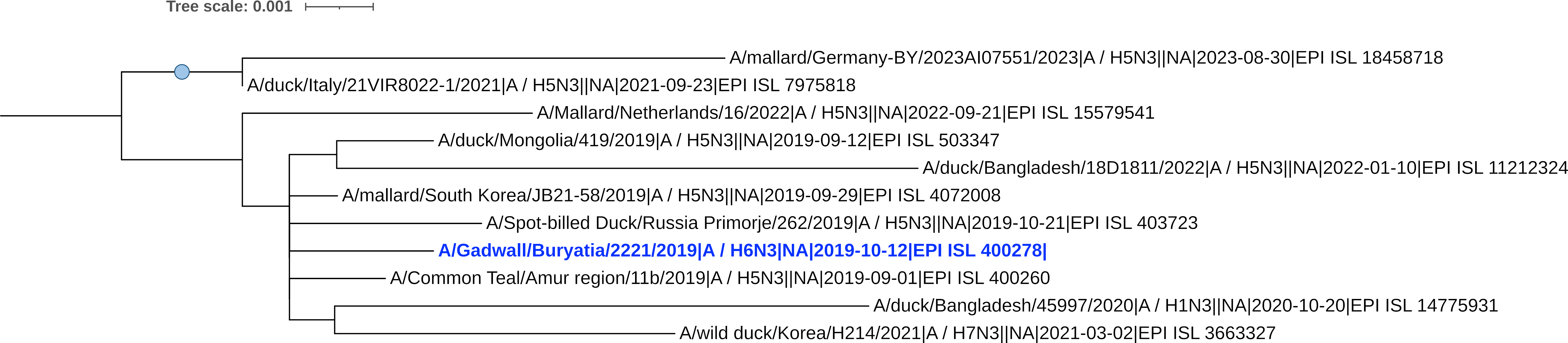

Supplement: Supplementary file 1 [file viruses-18-00761-s001.zip › Figure S4.png]

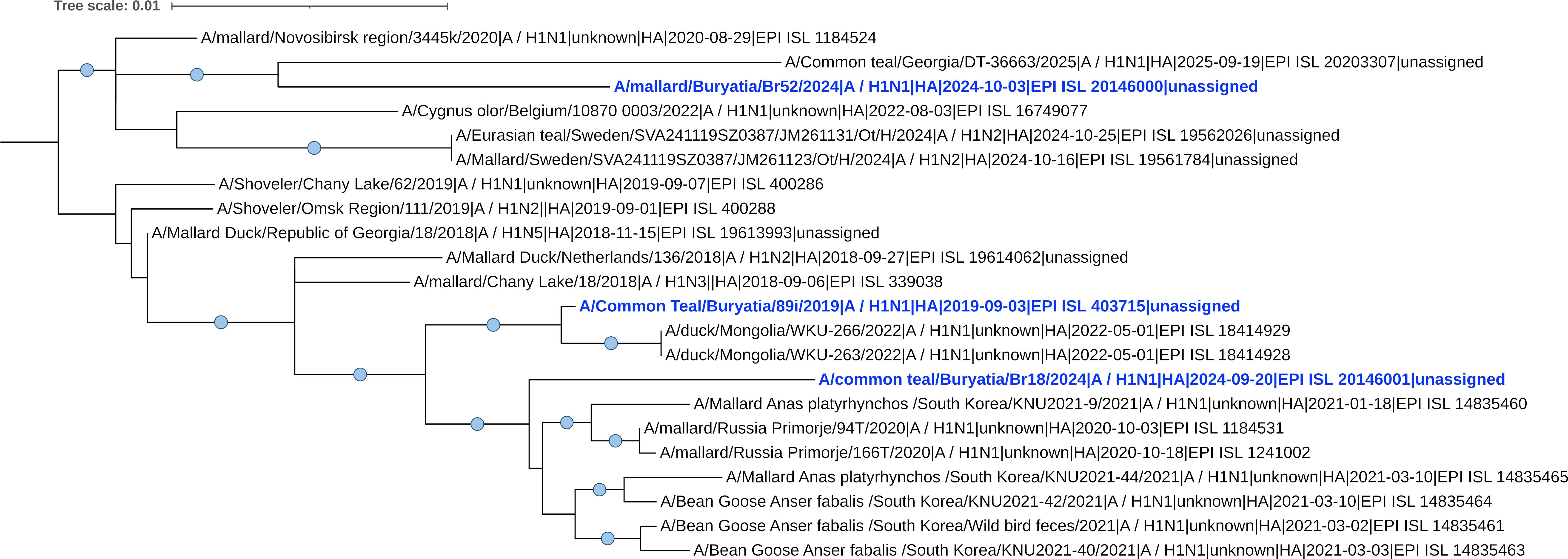

Supplement: Supplementary file 1 [file viruses-18-00761-s001.zip › Figure S5.png]
